# Supplementary material for: The relative impact of injury and deployment on mental and physical quality of life among military service members
Source: PLoS One. 2022 Sep 29;17(9):e0274973. doi: 10.1371/journal.pone.0274973 (PMC9522035; doi:10.1371/journal.pone.0274973)
Supplement: Attachment — Submitted filename: PONE-D-22-08419.pdf [file pone.0274973.s001.pdf]

PLOS ONE

The Relative Impact of Injury and Deployment on Mental and Physical Quality of Life among Military Service Members

--Manuscript Draft--

**Manuscript Number:**	PONE-D-22-08419
**Article Type:**	Research Article
**Full Title:**	The Relative Impact of Injury and Deployment on Mental and Physical Quality of Life among Military Service Members
**Short Title:**	Injury and Deployment on Mental and Physical Quality of Life among Military Service Members
**Corresponding Author:**	Claire Kolaja<br>Leidos Inc<br>San Diego, CA UNITED STATES
**Keywords:**	Quality of life; Injury; deployment; military
**Abstract:**	Background US service members injured in the recent conflicts in Iraq and Afghanistan were more likely to survive than those in previous conflicts because of advances in medicine and protective gear. However, there is limited research examining the long-term impact of injuries while deployed on physical and mental quality of life (QOL) among service members. Methods We used data from two time-points with an average follow-up period of 4.27 years (SD=2.13) from service members (n=118,054) to prospectively examine the association between deployment and injury status with QOL. Data were derived from the Millennium Cohort Study surveys (2001 to 2016) and linked with the Department of Defense Trauma Registry (DoD-TR) among a cohort of US service members from all branches and components. The primary predictor (a combination of deployment and injury status) was comprised of the following four categories: 1) not deployed, 2) deployed and not injured, 3) deployed and non-battle injured, and 4) deployed and battle injured. Demographic, military, psychological and behavioral health, and life stress factors were adjusted for in multivariable models. Outcomes of interest were physical and mental QOL from the Short-Form Health Survey for Veterans (VR-36) measured at ~4 year follow-up. Results Between group comparisons indicated that those deployed and battle-injured had the greatest decline in both mental (-3.82) and physical (-10.13) QOL scores over time (p < .05). While deployment and injury status were associated with poorer mental and physical QOL in adjusted models; only the association between deployment and injury status with physical QOL was clinically meaningful. In adjusted models, baseline mental and physical QOL explained most of the variance (23-25%) in follow-up mental and physical health QOL as compared to other covariates (e.g., injury and deployment, and other sociodemographic factors increased variance by ~5%). Conclusions Baseline QOL was the most significant predictor of later QOL, but those injured while deployed experienced significant and meaningful decrements to long-term physical QOL. This suggests that prevention and rehabilitation interventions should focus on improving physical health among injured service members to avoid long-term adverse effects.
**Order of Authors:**	Claire Kolaja
	Sheila F. Castañeda, PhD
	Susan I. Woodruff, PhD
	Rudolph P. Rull, PhD, MPH
	Richard F. Armenta, PhD, MPH
**Additional Information:**	
**Question**	**Response**
**Financial Disclosure**	The Millennium Cohort Study is funded through the Defense Health Program, U.S. Department of Veterans Affairs Office of Research and Development, and the U.S.

Powered by Editorial Manager® and ProduXion Manager® from Aries Systems Corporation

Enter a financial disclosure statement that describes the sources of funding for the work included in this submission. Review the submission guidelines for detailed requirements. View published research articles from *PLOS ONE* for specific examples.<br><br>This statement is required for submission and **will appear in the published article** if the submission is accepted. Please make sure it is accurate.<br><br>**Unfunded studies**<br>Enter: *The author(s) received no specific funding for this work.*<br><br>**Funded studies**<br>Enter a statement with the following details:<br>• Initials of the authors who received each award<br>• Grant numbers awarded to each author<br>• The full name of each funder<br>• URL of each funder website<br>• Did the sponsors or funders play any role in the study design, data collection and analysis, decision to publish, or preparation of the manuscript?<br>• **NO** - Include this sentence at the end of your statement: *The funders had no role in study design, data collection and analysis, decision to publish, or preparation of the manuscript.*<br>• **YES** - Specify the role(s) played.<br><br>* typeset	Department of Veterans Affairs Office of Patient Care Services under work unit no. 60002. The funding agency had no part in the study design, collection of the data, analysis of the data, or writing of manuscript. No financial disclosures were reported by the authors of this paper.
**Competing Interests**<br><br>Use the instructions below to enter a competing interest statement for this submission. On behalf of all authors, disclose any competing interests that could be perceived to bias this work—acknowledging all financial support and any other relevant financial or non-financial competing interests.<br><br>This statement is required for submission and **will appear in the published article** if the submission is accepted. Please make	The authors have declared that no competing interests exist.

sure it is accurate and that any funding sources listed in your Funding Information later in the submission form are also declared in your Financial Disclosure statement.<br><br>View published research articles from *PLOS ONE* for specific examples.<br><br>**NO authors have competing interests**<br><br>Enter: *The authors have declared that no competing interests exist.*<br><br>**Authors with competing interests**<br><br>Enter competing interest details beginning with this statement:<br><br>*I have read the journal's policy and the authors of this manuscript have the following competing interests: [insert competing interests here]*<br><br>* typeset	
**Ethics Statement**<br><br>Enter an ethics statement for this submission. This statement is required if the study involved:<br><br>• Human participants<br>• Human specimens or tissue<br>• Vertebrate animals or cephalopods<br>• Vertebrate embryos or tissues<br>• Field research<br><br>Write "N/A" if the submission does not require an ethics statement.<br><br>General guidance is provided below. Consult the submission guidelines for detailed instructions. **Make sure that all information entered here is included in the Methods section of the manuscript.**	The study was approved by the Naval Health Research Center IRB (protocol number NHRC.2000.0007).

**Format for specific study types**<br><br>**Human Subject Research (involving human participants and/or tissue)**<br>• Give the name of the institutional review board or ethics committee that approved the study<br>• Include the approval number and/or a statement indicating approval of this research<br>• Indicate the form of consent obtained (written/oral) or the reason that consent was not obtained (e.g. the data were analyzed anonymously)<br><br>**Animal Research (involving vertebrate animals, embryos or tissues)**<br>• Provide the name of the Institutional Animal Care and Use Committee (IACUC) or other relevant ethics board that reviewed the study protocol, and indicate whether they approved this research or granted a formal waiver of ethical approval<br>• Include an approval number if one was obtained<br>• If the study involved *non-human primates*, add *additional details* about animal welfare and steps taken to ameliorate suffering<br>• If anesthesia, euthanasia, or any kind of animal sacrifice is part of the study, include briefly which substances and/or methods were applied<br><br>**Field Research**<br><br>Include the following details if this study involves the collection of plant, animal, or other materials from a natural setting:<br>• Field permit number<br>• Name of the institution or relevant body that granted permission	
**Data Availability**<br><br>Authors are required to make all data underlying the findings described fully available, without restriction, and from the time of publication. PLOS allows rare exceptions to address legal and ethical concerns. See the PLOS Data Policy and FAQ for detailed information.	No - some restrictions will apply

A Data Availability Statement describing where the data can be found is required at submission. Your answers to this question constitute the Data Availability Statement and **will be published in the article**, if accepted.<br><br>**Important:** Stating 'data available on request from the author' is not sufficient. If your data are only available upon request, select 'No' for the first question and explain your exceptional situation in the text box.<br><br>Do the authors confirm that all data underlying the findings described in their manuscript are fully available without restriction?	
**Describe where the data may be found in full sentences. If you are copying our sample text, replace any instances of XXX with the appropriate details.**<br><br>• If the data are **held or will be held in a public repository**, include URLs, accession numbers or DOIs. If this information will only be available after acceptance, indicate this by ticking the box below. For example: *All XXX files are available from the XXX database (accession number(s) XXX, XXX.*).<br>• If the data are all contained **within the manuscript and/or Supporting Information files**, enter the following: *All relevant data are within the manuscript and its Supporting Information files.*<br>• If neither of these applies but you are able to provide **details of access elsewhere**, with or without limitations, please do so. For example:<br><br>*Data cannot be shared publicly because of [XXX]. Data are available from the XXX Institutional Data Access / Ethics Committee (contact via XXX) for researchers who meet the criteria for access to confidential data.*<br><br>*The data underlying the results presented in the study are available from (include the name of the third party*	Data Availability Statement: The datasets analyzed during the current study are not publicly available due to institutional regulations protecting service member survey responses but are available from the corresponding author on reasonable request (may require data use agreements to be developed).

and contact information or URL).<br>• This text is appropriate if the data are owned by a third party and authors do not have permission to share the data.<br><br>* typeset	
Additional data availability information:	

Manuscript
Click here to access/download;Manuscript;Manuscript_11MAR2022.docx

Word count: 3,643; tables/figures: 5

The Relative Impact of Injury and Deployment on Mental and Physical Quality of Life among Military Service Members

Claire Kolaja*, MPH[1,2], Sheila F. Castañeda, PhD[1,2], Susan I. Woodruff, PhD[3], Rudolph P. Rull, PhD, MPH[2], Richard F. Armenta, PhD, MPH[4] for the Millennium Cohort Study Team

[1]Leidos Inc., San Diego, CA

[2]Deployment Health Research Department, Naval Health Research Center, San Diego, CA

[3]San Diego State University, School of Social Work, 5500 Campanile Drive, HH203, San Diego, CA 92182

[4]Department of Kinesiology, College of Education, Health and Human Services, California State University, San Marcos, San Marcos, CA

*Correspondence author

Email: Claire.a.kolaja.ctr@mail.mil (CK)

Specific authors contributions:

CK – Methodology, Formal analysis, Investigation, Data Curation, Writing – Original draft, Writing – Review & Editing, Project administration

SC – Methodology, Formal analysis, Investigation, Writing – Original draft, Writing – Review & Editing, Project administration

SW – Conceptualization, Writing – Original draft, Writing – Review & Editing

RR – Writing – Review & Editing, Project administration

RA – Conceptualization, Writing – Original draft, Writing – Review & Editing

Italicize the statistical symbols according to APA

Abstract

US service members injured in the recent conflicts in Iraq and Afghanistan were more likely to survive than those in previous conflicts because of advances in medicine and protective gear. However, there is limited research examining the long-term impact of injuries while deployed on physical and mental quality of life (QOL) among service members. We used data from two time-points with an average follow-up period of 4.27 years ($SD=2.13$) from service members ($n=118,054$) to prospectively examine the association between deployment and injury status with QOL. Data were derived from the Millennium Cohort Study surveys (2001 to 2016) and linked with the Department of Defense Trauma Registry (DoD-TR) among a cohort of US service members from all branches and components. The primary predictor (a combination of deployment and injury status) was comprised of the following four categories: 1) not deployed, 2) deployed and not injured, 3) deployed and non-battle injured, and 4) deployed and battle injured. Demographic, military, psychological and behavioral health, and life stress factors were adjusted for in multivariable models. Outcomes of interest were physical and mental QOL from the Short-Form Health Survey for Veterans (VR-36) measured at ~4 year follow-up. Between group comparisons indicated that those deployed and battle-injured had the greatest decline in both mental (-3.82) and physical (-10.13) QOL scores over time ($p < .05$). While deployment and injury status were associated with poorer mental and physical QOL in adjusted models; only the association between deployment and injury status with physical QOL was clinically meaningful. In adjusted models, baseline mental and physical QOL explained most of the variance (23-25%) in follow-up mental and physical health QOL as compared to other covariates (e.g., injury and deployment, and other sociodemographic factors increased variance by ~5%). Baseline QOL was the most significant predictor of later QOL, but those injured while deployed experienced significant and meaningful decrements to long-term physical QOL. This suggests that prevention and rehabilitation interventions should focus on improving physical health among injured service members to avoid long-term adverse effects.

Introduction

care (1). Pls, correct all these typos trough out the MS
conflicts (2).

The survival rate of those combat-injured in Operation Enduring Freedom/Operation Iraqi Freedom/Operation New Dawn (OEF/OIF/OND) was the highest in the past 100 years, primarily due to advanced protective gear and rapid effective medical care.(1) Approximately 56,000 military personnel were combat-injured in OEF/OIF/OND conflicts.(2) Both the causes and outcomes of these combat injuries were distinctive from previous conflicts, with exposures to improvised explosive devices (IEDs) resulting in a high risk of repeated mild and more severe traumatic brain injuries (TBI).(3) In addition to physical trauma, mental health outcomes, such as posttraumatic stress disorder (PTSD) and depression, have also received widespread attention due to their relatively high prevalence among OEF/OIF/OND veterans.(4)

While the financial cost of compensating and caring for the wounded from these conflicts will be staggering (estimated over $2 trillion),(5) there are other health impacts outside of objective injury and disease indicators that warrant attention. Recent research on the impact of injury and disease has moved beyond the traditional biomedical model, to more ecological models of health and well-being, which recognize that objective disease and disability status alone is insufficient for capturing the long-term impact of illness/injury at the individual level.(6) Quality of life (QOL) is a concept that, when measured well, is thought to shed light on both the objective and subjective experience during and after illness and injury. In fact, consideration of mental and physical wellbeing and QOL is now recognized as an integral component of providing healthcare.(7)

QOL is typically measured through both physical health (i.e., functioning, physical limitations and bodily pain) and mental health (i.e., perceived social functioning, mental health, and general well-being).(8) QOL measures provide additional contextual and functional information that health care providers may use to have a broader picture of an individual's life circumstances,(6) which may be useful when assessing the need for care and rehabilitation.(9, 10)

Research suggests that a range of demographic and psychosocial factors, such as older age, physical health,(11, 12) mental health symptoms, unhealthy behaviors (e.g., smoking, poor sleep),(13-16) and life stressors (17) are associated with lower QOL. Additionally, research among service members and veterans has shown that service-related factors, (e.g., deployment, service branch, pay grade, component, and combat injury), pre-existing physical health conditions (e.g., asthma and hypertension), and mental health factors (e.g., PTSD) have been found to be predictive of QOL.(12, 16, 18) Combat injury, specifically, has been assessed as a QOL correlate to some degree (4, 10) but these studies have methodological limitations (e.g., cross-sectional analyses or convenient sampling), or focused on a specific type of trauma such as limb loss or concussion. A study of health-related QOL among those wounded in combat showed depression and PTSD were associated with lower QOL.(18) Others have reported that deployment (i.e., irrespective of combat and whether an injury was acquired) may be associated with decreased psychological health over time.(19, 20) However, there is a dearth of information on how deployment and injury status influence QOL independently of one another. It is important to discern the differential and combined effects of both deployment and injury on health-related QOL to inform future prevention and intervention efforts aimed at improving service member readiness and well-being. Therefore, the primary objective of this study was to

understand the relative contribution of deployment and injury status (both battle and non-battle) on service member long-term physical and mental QOL.

Materials and methods

Participants, instruments, procidement, and data analyses

The Millennium Cohort Study is the largest longitudinal cohort study of active duty, Reserve/National Guard military personnel and veterans. Launched in 2001, the Millennium Cohort Study has enrolled over 250,000 participants. All participants provided written informed consent at enrollment and were enrolled into five distinct panels between 2001 and 2021 after being randomly selected from active and reserve rosters. Study participants complete comprehensive self-reported surveys every 3 to 5 years that assess factors such as health and behavioral measures, QOL, and military service factors. External administrative records can be linked to participant survey data to supplement exposure or outcome data. Additional information about study enrollment, methodology, and response rates has been previously published.(21, 22) The study was approved by the Naval Health Research Center IRB (protocol number NHRC.2000.0007).

The study sample consisted of 118,054 Millennium Cohort Study participants who met eligibility criteria. For this study, participant records from the first four panels (enrolled from 2001- 2013) were linked to Department of Defense Trauma Registry (DoD-TR) data from January 2002 to July 2016.

The main inclusion criterion for this analysis was completion of two Millennium Cohort surveys. For study participants identified in the DoD-TR, the surveys must have been completed before and after the injury occurrence. In addition, participants must have completed the QOL measure on the two surveys. Overall, 1,320 (<1%) Millennium Cohort Study participants were identified in the DoD-TR database between 2002 and 2016 of whom 30 were deceased from the injury and two were missing information on injury location (battle versus non-battle) so were excluded. Those not injured were categorized using administrative deployment dates from the Contingency Tracking System (CTS). Per nomenclature used in recent articles (23, 24), a four level variable was created, which included the following categories: (a) deployed and battle injured (DBI), (b) deployed and non-battle injured (DNBI), (c) deployed and not injured (DNI), and (d) not deployed (ND).

Among the 135,854 participants identified based on the above mentioned eligibility criteria, 17,800 (13%) were excluded due to missing covariate information at baseline, resulting in a final study sample of 118,054 participants.

QOL was assessed using the Short-Form Health Survey for Veterans (SF-36V),(25) a QOL measure included on every Millennium Cohort Study survey through the 2014 cycle. The SF-36V is a widely used, standardized QOL measure that relies on participants' self-report of health status and functioning over the past 4 weeks. It contains 36 questions that measure physical and mental health across eight domains: physical functioning, vitality, bodily pain, general health perceptions, physical role functioning, emotional role functioning, social role functioning, and mental health. Domains are combined into physical QOL (physical component summary, or PCS) and mental QOL (mental component summary, or MCS) scales which average the physical and mental relevant questions, respectively. The MCS and PCS scores are computed to compare to US population normative values of 50 (SD = 10) where higher scores indicate better QOL.(25)

include a flow-chart figure with the procidement of participants according to inclusion and exclusion criteria

Injury information for Millennium Cohort Study participants was obtained from the DoD-TR in July 2016 for all injuries among Millennium Cohort Study participants that occurred before this date. The DoD-TR is a registry maintained by DoD Center of Excellence for Trauma that collects and maintains records of injuries that occur among OIF/OEF/OND deployed military service members from the point of injury to final disposition.(26) The registry was initially created to improve battlefield care and assess quality of care. Using DoD-TR data, participants were categorized as battle or not-battle injured; the date of the injury was used to identify the most proximal pre- and post- Millennium Cohort Study surveys to use for analysis. Participants not in the DoD-TR were further categorized as deployed and not injured, and not deployed personnel using deployment dates in support of OIE/OIF/OND obtained from the CTS.

All covariates (i.e., age, marital status, education, component, service branch, pay grade, number of deployments, health behaviors, mental health, and life stressors) were assessed at the baseline survey. Sex, age at baseline, race and ethnicity (i.e., non-Hispanic White, non-Hispanic Black, and other), military component (i.e., active duty, Reserve/National Guard), service branch (i.e., Army, Air Force, Navy/Coast Guard, Marine Corps), and pay grade (i.e., enlisted, officer) were obtained from the Defense Manpower Data Center (DMDC). The Other race and ethnicity category included Hispanic/Latino, Asian American or Pacific Islander, American/Alaskan Indian and other (unspecified) service members. Marital status and education were self-reported and backfilled if missing with DMDC data. Dates for deployments in support of OIE/OIF/OND provided from CTS were used to determine the number of deployments before baseline.

Based on National Sleep Foundation recommendations sleep duration was categorized as less than 6, 6, 7–9, and more than 9 hours using self-reported average number of hours slept daily in the past month.(27) Smoking status was based on responses to two questions regarding smoking at least 100 cigarettes in their lifetime and cessation such that never smokers smoked less than 100 cigarettes, and those who smoked at least 100 cigarettes were classified as former smokers if they reported quitting successfully, or current smokers, if they had not quit.(28) Depression symptoms (no/yes) were assessed using the PHQ depression scale (PHQ-8) based on DSM-IV-TR criteria. Participants screened positive for depression symptoms based on the following criteria: responded “more than half the days” or “nearly every day” to at least 5 items with at least one item endorsed being depressed mood or anhedonia.(29) PTSD symptoms (no/yes) were assessed using the PTSD Checklist – Civilian Version (PCL-C). The measure consists of 17 items that evaluate PTSD symptoms during the previous month. PTSD symptoms were determined based using the criteria as defined in the DSM-IV (30) for participants who reported “moderate” or greater on at least one intrusion symptom, three avoidance symptoms, and two hyperarousal symptoms. Chronic life stressors were categorized as none, one, or two or more based on the number of endorsed stressful life events experienced in the past three years including the following six events: divorce, financial problems, physical assault, sexual assault, and illness or death of a loved one. (31) Time between the two surveys was calculated in years.

Statistical analyses

Means and standard deviations for baseline and follow-up MCS, PCS and change in MCS and PCS were calculated by injury/deployment status (Table 1). The mean differences of baseline, follow-up, and change over time in MCS and PCS between the four injury/deployment groups were compared using Tukey post hoc tests. Descriptive statistics (frequencies and percentages or means and standard deviation) were calculated for all covariates by the injury/deployment status (Table 1). Collinearity was assessed among the predictors and was tested with a variance inflation factor threshold of ≥4.

Hierarchical linear regression was employed to examine the association between injury/deployment status with QOL. For both MCS and PCS three models were run: Model 1 included baseline MCS for the MCS outcome and baseline PCS for the PCS outcome; Model 2 added the injury/deployment status to Model 1; Model 3 added time between the surveys, demographic factors (i.e., age, sex, race and ethnicity, marital status, education), military factors (i.e., component, service branch, pay grade and number of deployments), and other factors (i.e., sleep duration, smoking status, major depression, PTSD, life stressors) to Model 2. R^2 and root mean square error (MSE) were reported for each model (see Tables 2 and 3 for MCS and PCS, respectively). Statistical analyses were performed using SAS software version 9.4.(32)

Results

Descriptive Analyses

Among the 118,054 eligible participants, 95,921 ND (81.3%), 21,745 DNI (18.4%), and 388 were injured (0.3%; 197 DNBI and 191 DBI). The sample was 69.3% male, and 75.8% non-Hispanic White with a mean age of 31.9 years at baseline (SD = 9.0; Table 1). Most participants were married (59.0%), had less than a college degree (55.8%), served on active duty (60.7%), were enlisted personnel (77.6%), and almost half served in the Army (45.4%). The majority did not screen positive for depression or PTSD symptoms (96.0% and 93.8%, respectively), reported recommended health behaviors (e.g., 47.5% an average of 7-9 hours of sleep, 59.0% never smoked cigarettes) and reported no life stressors (58.7%) at baseline. Compared to those who were DNI, DBI were more likely to be male, active duty, enlisted pay grade, and serving in the Army. The average time between the baseline and follow-up surveys was 4.3 years (SD=2.1) and the average time between injury and follow-up survey was 2.3 (SD=2.0). Fully adjusted models adjusted for these variables (see Tables 2 and 3).

Table 1: Demographic, behavioral, and military characteristics by injury status among Millennium Cohort participants, n=118,054

	Not deployed (n=95,921)	Deployed and not injured (n=21,745)	Deployed and injured	
			Nonbattle (n=197)	Battle (n=191)
**Variable**	**N (%)**	**N (%)**	**N (%)**	**N (%)**
Time between baseline and follow-up surveys, in years (Mean, SD)	4.20 (2.22)	4.54 (1.62)	5.96 (3.29)	5.46 (3.03)
**MCS***				
Baseline (Mean, SD)	51.21 (10.24)	52.40 (8.58)	52.31 (8.85)	50.93 (10.10)
Change score (Mean, SD)	-1.43 (10.66)	-1.57 (10.20)	-3.35 (11.95)	-3.82 (13.20)
Follow-up (Mean, SD)	49.78 (11.27)	50.83 (10.08)	48.96 (12.36)	47.11 (13.00)
**PCS****				
Baseline (Mean, SD)	53.32 (7.84)	55.23 (5.97)	54.20 (6.36)	54.35 (6.68)
Change score (Mean, SD)	-1.13 (8.22)	-1.76 (7.68)	-8.80 (11.78)	-10.13 (11.78)
Follow-up (Mean, SD)	52.19 (8.49)	53.47 (7.39)	45.40 (11.48)	44.23 (11.05)
**Covariates**				
Age, in years (mean, SD)	32.56 (9.23)	29.27 (7.06)	29.19 (7.23)	28.16 (6.07)
Sex				
Male	64,736 (67.49)	16,704 (76.82)	162 (82.23)	178 (93.19)
Female	31,185 (32.51)	5,041 (23.18)	35 (17.77)	13 (6.81)
Race and Ethnicity				
Non-Hispanic Black	10,687 (11.14)	2,121 (9.75)	27 (13.71)	17 (8.90)

Non-Hispanic White	72,541 (75.63)	16,653 (76.58)	146 (74.11)	154 (80.63)
Other (including Hispanic, Asian/Pacific Islander, American/Alaskan Indian and other)	12,693 (13.23)	2,971 (13.66)	24 (12.18)	20 (10.47)
Marital Status				
Single	26,859 (28.00)	7,514 (34.56)	60 (30.46)	55 (28.80)
Married	57,222 (59.66)	12,240 (56.29)	112 (56.85)	125 (65.45)
Separated	11,840 (12.34)	1,991 (9.16)	25 (12.69)	11 (5.76)
Education				
Less than college	53,066 (55.32)	12,517 (57.56)	133 (67.51)	134 (70.16)
College degree	42,855 (44.68)	9,228 (42.44)	64 (32.49)	57 (29.84)
Component				
Reserve/ National Guard	39,583 (41.27)	6,656 (30.61)	54 (27.41)	56 (29.32)
Active duty	56,338 (58.73)	15,089 (69.39)	143 (72.59)	135 (70.68)
Service Branch				
Army	43,932 (45.80)	9,404 (43.25)	147 (74.62)	165 (86.39)
Navy or Coast Guard	18,966 (19.77)	2,777 (12.77)	13 (6.60)	3 (1.57)
Marine	7,934 (8.27)	1,326 (6.10)	15 (7.61)	14 (7.33)
Air Force	25,089 (26.16)	8,238 (37.88)	22 (11.17)	9 (4.71)
Pay Grade				
Enlisted	75,036 (78.23)	16,258 (74.77)	165 (83.76)	160 (83.77)
Officer	20,885 (21.77)	5,487 (25.23)	32 (16.24)	31 (16.23)
Number deployments (Mean, SD)	0.39 (0.85)	0.71 (1.32)	0.45 (0.70)	0.40 (0.64)
Average hours of sleep				
Less than 6 hours	17,734 (18.49)	3,861 (17.76)	49 (24.87)	45 (23.56)
6 hours	29,610 (30.87)	6,957 (31.99)	61 (30.96)	74 (38.74)
7-9 hours	45,535 (47.47)	10,440 (48.01)	79 (40.10)	65 (34.03)
More than 9 hours	3,042 (3.17)	487 (2.24)	8 (4.06)	7 (3.66)
Smoking status				
Never	56,002 (58.38)	13,470 (61.95)	107 (54.31)	94 (49.21)
Former	23,695 (24.70)	4,611 (21.20)	46 (23.35)	46 (24.08)
Current	16,224 (16.91)	3,664 (16.85)	44 (22.34)	51 (26.70)
Depression symptoms (yes)	4,209 (4.39)	465 (2.14)	6 (3.05)	7 (3.66)
PTSD symptoms (yes)	6,477 (6.75)	774 (3.65)	6 (3.05)	13 (6.81)
Life Stressors				
None	54,266 (56.57)	14,829 (68.19)	118 (59.90)	120 (62.83)
One	35,843 (26.94)	4,786 (22.01)	54 (27.41)	51 (26.70)
2 or more	15,812 (16.48)	2,130 (9.80)	25 (12.69)	20 (10.47)

MCS - Mental Composite Summary; PCS - Physical Composite Summary; MCS and PCS are derived from the Short-Form Health Survey for Veterans measure

*Mean MCS at baseline varied significantly by deployment and injury status (p-value < .0001). Pair wise comparisons using Tukey multiple comparison procedure revealed one significant (p-value < 0.05) group difference in the mean MCS at baseline between ND and DNI. Mean change in MCS between baseline and follow-up varied significantly by injury/deployment status (p-value < .0001). Pair wise comparisons using Tukey multiple comparison procedure revealed two significant (p-value < 0.05) group differences in the change in MCS between 1) DBI and ND, and 2) DBI and DNI. Mean MCS at follow-up varied significantly by injury/deployment status. Pair wise comparisons using Tukey multiple comparison procedure revealed three significant differences (p-value < 0.05) in mean MCS at follow-up between 1) ND and DNI, 2) DBI and DNI, and 3) DBI and ND.

** Mean PCS scores at baseline varied significantly by deployment and injury status (p-value < .0001). Pair wise comparisons using Tukey multiple comparison procedure revealed one significant (p-value < 0.05) group difference in the mean PCS between those DNI and ND. Mean change in PCS between baseline and follow-up varied significantly by injury/deployment status (p-value < .0001). Pair wise comparisons using Tukey multiple comparison procedure revealed significant (p-value < 0.05) group differences in the change in PCS between all groups except those DNBI and DBI. Mean PCS at follow-up varied significantly by injury/deployment status. Pair wise comparisons using Tukey multiple comparison procedure revealed significant difference (p-value < 0.05) in mean PCS at follow-up between all comparisons except between DNBI and DBI.

Smoking status – defined as never and former/current based off of responses to questions regarding smoking at least 100 cigarettes in their lifetime and successfully quitting smoking cigarettes.

Depression symptoms – assessed using the PHQ depression scale (PHQ-8) based on DSM-IV-TR criteria. Participants screened positive for depression based on the following criteria: responded “more than half the days” or “nearly every day” to at least 5 items with at least one item endorsed being depressed mood or anhedonia.
PTSD symptoms – assessed using the PTSD Checklist – Civilian Version. The measure consists of 17 items which evaluate PTSD symptoms during the previous month. A positive PTSD screen was determined based using the criteria as defined in the Diagnostic and Statistical Manual of Mental Disorders, Fourth Edition, Text Revision.
Life stressors – based on the number of endorsed stressful life experiences in the past 3 years including endorsement of divorce, financial problems, physical assault, sexual assault, and illness or death of a loved.

Between group differences in QOL

Mean MCS at baseline varied by deployment and injury status (mean of 51.2 for ND, 52.4 for DNI, 52.3 for DNBI, and 50.9 for DBI; p-value < .0001; Table 1 and Figure 1) and pairwise comparisons using Tukey multiple comparison procedures revealed a significant (p-value < 0.05) group difference between ND and DNI’s mean scores. Mean change in MCS between baseline and follow-up also varied significantly by deployment and injury status (mean change for ND -1.4, DNI -1.6, DNBI -3.4, and DBI -3.8; p-value < .0001; Table 1 and Figure 1). Pairwise comparisons revealed two significant (p-value < 0.05) group differences in the change in MCS between those DBI and the two non-injured groups. Finally, mean MCS at follow-up varied significantly by deployment and injury status (Table 1 and Figure 1) and pairwise comparisons revealed three significant differences (p-value < 0.05) in mean MCS at follow-up between 1) ND and DNI, 2) DNI and DBI, and 3) ND and DBI.

Similar trends were observed for PCS with more pronounced differences observed by deployment and injury status. Mean PCS at baseline varied by deployment and injury status (mean of 53.3 for ND, 55.2 for DNI, 54.2 for DNBI, and 54.4 for DBI; p-value < .0001; Table 1 and Figure 2) and pairwise comparisons revealed a significant (p-value < 0.05) group difference between ND and DNI’s mean baseline PCS. Mean change in PCS between baseline and follow-up varied significantly by deployment and injury status (mean change for ND -1.1, DNI -1.8, DNBI -8.8, and DBI -10.1; p-value < .0001; Table 1 and Figure 2) with significant (p-value < 0.05) group differences found between all groups except the two injured groups. Additionally, mean PCS at follow-up varied significantly by deployment and injury status (Table 1 and Figure 2) where pairwise comparisons again revealed significant difference (p-value < 0.05) between all groups except between the two injured groups.

Modeling Follow-up Mental Health QOL (MCS)

Table 2 lists results from three hierarchical linear regression models for follow-up MCS. Model 1, which only included baseline MCS, explained almost 25.0% of the variance in follow-up MCS ($R^2 = 0.25$; root MSE = 9.61). Although the addition of deployment and injury status in Model 2 did not increase the variance in follow-up MCS explained, deployment and injury status were significantly associated with follow-up MCS. Compared to deployed and not injured service members, DNBI, DBI and ND were associated with lower follow-up MCS (average of 2.90, 1.82 and 0.39, respectively). When all covariates were added in Model 3, the variance explained increased by 4.6% ($R^2 = 0.29$; root MSE = 9.31). DBI and ND status were associated with lower follow-up MCS (average of 1.86, and 0.32, respectively). Additional factors associated with lower follow-up MCS included: time between surveys, female sex, other race and ethnicity, Reserve/National Guard service, Marine Corp (compared to Army), number of deployments, less than 7 or more than 9 average hours of sleep, former or current cigarette smoking, depression symptoms, PTSD symptoms, and life stressors. Conversely, the following characteristics were significantly associated with a higher follow-up MCS: higher baseline MCS

and PCS, older age, non-Hispanic Black race and ethnicity, Navy/Coast Guard or Air Force service (compared to Army), and officer pay grade.

Table 2: Associations between main exposure and covariates with Mental Composite Scores (MCS) among Millennium Cohort participants, n=118,054

Variable	Model 1 Estimate (SE)	Model 2 Estimate (SE)	Model 3 Estimate (SE)
**Baseline MCS**	0.554 (0.003) **	0.553 (0.003)**	0.468 (0.004)**
**Baseline PCS**			0.174 (0.004)**
**Injury Category (ref: Deployed and not injured)**			
Deployed and battle injured		-2.904 (0.698)**	-1.863 (0.677)*
Deployed and nonbattle injured		-1.817 (0.687)*	-0.829 (0.667)
Not deployed		-0.388 (0.072)**	-0.321 (0.073)**
**Time between surveys, in years**			-0.254 (0.013)**
**Age (5-year change)**			0.552 (0.019)**
**Female sex (ref: Male)**			-0.467 (0.064)**
**Race and Ethnicity (ref: Non-Hispanic White)**			
Non-Hispanic Black			0.981 (0.091)**
Other (including Hispanic, Asian/Pacific Islander, American/Alaskan Indian and other)			-0.172 (0.081)*
**Marital Status* (ref: Single)**			
Married			0.121 (0.068)
Separated			0.233 (0.105)*
**College Degree Education* (ref: Some college or less)**			0.013 (0.070)
**Reserve/National Guard Component* (ref: Active Duty)**			-0.442 (0.061)**
**Service Branch* (ref: Army)**			
Navy/Coast Guard			0.507 (0.077)**
Marine			-0.348 (0.108)*
Air Force			1.611 (0.068)**
**Officer Pay Grade* (ref: Enlisted)**			0.447 (0.083)**
**Number Deployments (cumulative)**			-0.528 (0.029)**
**Average hours of sleep (ref: 7-9 hours)**			
< 6 hours			-0.866 (0.082)**
6 hours			-0.382 (0.064)**
9 < hours			-0.619 (0.164)*
**Smoking status (ref: never)**			
Former			-0.439 (0.068)**
Current			-0.618 (0.079)**
**Depression symptoms (ref: no)**			-0.472 (0.170)*
**PTSD symptoms (ref: no)**			-2.152 (0.145)**
**Life Stressors (ref: none)**			
One			-0.464 (0.069)**
2 or more			-1.287 (0.090)**
**R^2**	0.248	0.248	0.294
**Root MSE**	9.605	9.603	9.308

* <0.05 ** <0.0001

Modeling Follow-up Physical Health QOL (PCS)

The results from the three hierarchical linear regression models for follow-up PCS are listed in Table 3. Model 1 examined baseline PCS on follow-up PCS and explained almost 23%

of the variance in follow-up PCS (R^2 = 0.23; root MSE = 7.33). With the addition of deployment and injury status in Model 2, the variance explained increased by 0.03%. In addition, deployment and injury status was significantly associated with follow-up PCS, with DBI, DNBI, and ND associated with lower follow-up PCS (average of 8.78, 7.52 and 0.28, respectively). Model 3, which included all covariates, additionally increased the variance explained by 4.6% (R^2 = 0.28; root MSE = 7.10). In the fully adjusted Model 3, DBI, DNBI, and ND were again associated with lower follow-up PCS (average of 7.94, 6.58, and 0.16, respectively). Other characteristics significantly associated with lower follow-up PCS included: time between surveys, older age, female sex, non-Hispanic Black race and ethnicity, currently married or separated marital status, more deployments, less than 7 average hours of sleep, former or current cigarette smoking, PTSD symptoms, and any life stressors. Factors in Model 3 associated with a higher follow-up PCS include: higher baseline MCS and PCS, college degree, Reserve/National Guard service, Navy or Coast Guard, Marine Corps or Air Force service (compared to Army), and officer pay grade (p-value < 0.001).

Table 3: Associations between main exposure and covariates with Physical Composite Scores (PCS) among Millennium Cohort participants, n=118,054

Variable	Model 1 Estimate (SE)	Model 2 Estimate (SE)	Model 3 Estimate (SE)
**Baseline PCS**	0.526 (0.003)**	0.525 (0.003)**	0.476 (0.003)**
**Baseline MCS**			0.077 (0.003)**
**Injury Category (ref: Deployed and not injured)**			
Deployed and battle injured		-8.784 (0.532)**	-7.937 (0.516)**
Deployed and nonbattle injured		-7.529 (0.524)**	-6.580 (0.508)**
Not deployed		-0.278 (0.055)**	-0.162 (0.055)*
**Time between surveys, in years**			-0.386 (0.010)**
**Age (5-year change)**			-0.448 (0.015)**
**Female sex (ref: Male)**			-0.109 (0.049)*
**Race and Ethnicity (ref: Non-Hispanic White)**			
Non-Hispanic Black			-0.314 (0.070)**
Other (including Hispanic, Asian/Pacific Islander, American/Alaskan Indian and other)			-0.112 (0.062)
**Marital Status* (ref: Single)**			
Married			-0.366 (0.052)**
Separated			-0.454 (0.080)**
**College Degree Education* (ref: Some college or less)**			0.441 (0.054)**
**Reserve/National Guard Component* (ref: Active Duty)**			0.758 (0.047)**
**Service Branch* (ref: Army)**			
Navy/Coast Guard			1.079 (0.059)**
Marine			0.498 (0.082)**
Air Force			0.622 (0.052)**
**Officer Pay Grade* (ref: Enlisted)**			0.941 (0.063)**
**Number Deployments (cumulative)**			-0.391 (0.022)**
**Average hours of sleep (ref: 7-9 hours)**			
< 6 hours			-0.960 (0.062)**
6 hours			-0.307 (0.049)**
9 < hours			-0.101 (0.125)
**Smoking status (ref: never)**			
Former			-0.231 (0.052)**
Current			-0.601 (0.060)**

**Depression symptoms (ref: no)**			0.041 (0.130)
**PTSD symptoms (ref: no)**			-0.308 (0.110)*
**Life Stressors (ref: none)**			
One			-0.390 (0.053)**
2 or more			-1.025 (0.069)**
**R^2**	0.227	0.230	0.276
**Root MSE**	7.330	7.315	7.095
* <0.05 ** <0.0001			

Discussion

This study examined longitudinal changes in mental and physical QOL by deployment and injury status in a large sample of service members. Results showed that although mental and physical QOL decreased for all deployment and injury subgroups, physical QOL significantly and meaningfully decreased among both injured groups, regardless of injury type (i.e., battle or non-battle). The average decreases on the PCS exceed previously identified clinically meaningful difference of 2.5units on the SF-36 QOL scale.(33) While some between-group differences on the MCS were statistically significant, they did not meet the threshold for clinically meaningful differences (less than 2 points observed difference). As these differences on the PCS are clinically impacted after injury during deployment, regardless of injury type, additional services could be offered to improve physical-related QOL of service members that return from deployment with an injury.

Deployment and injury status was significantly associated with both follow-up QOL outcomes when adjusting for covariates with the worse QOL scores observed for those who deployed and battle injured (DBI). Similar results were observed among a sample of Dutch OEF/OIF veterans (n=188), where service members with battle casualties reported significantly lower QOL five years later compared to non-injured.(34) However, only an overall QOL score was assessed so direct comparisons cannot be made with mental and physical health constructs. Additionally, one study that compared QOL between battle and non-battle injured Dutch service members found that those injured in battle are most at risk.(35) Battle injuries that occurred in OEF/OIF/OND were primarily caused by explosive devices (36, 37); and those DBI (versus DNBI) were more likely to have multiple and severe TBIs.(38) Although not assessed in this study, previous literature has shown that TBIs have a negative effect on long-term quality of life.(39)

We observed that those who deployed and had a non-battle injury (DNBI) were more likely to have lower QOL than those who deployed and were not injured (DNI). Few studies have examined QOL after this type of injury.(40) Similar to our findings, a study of Dutch OEF/OIF deployed personnel (n=223) found significant decreases in physical QOL and no observed difference in mental QOL among those injured with a non-battle injury compared to uninjured deployed service members.(24) This study was a cross-sectional design, possibly unable to assess the long-term impact for injury. The primary causes of non-battle injuries among OEF/OIF/OND deployers has been identified as sports/athletics, fall/jumps, or lifting heavy gear.(40, 41) Although there has been a focus on battle injuries, non-battle injuries account for approximately a third of injuries sustained in OEF/OIF/OND, leading to attrition, increased medical costs, and affecting readiness of deployed units.(42)

In our study, we also found that those who did not deploy (ND) also had lower QOL compared to those who deployed and not injured (DNI), although this was the smallest observed difference (less than -0.3). A similar study that compared personnel deployed to the Gulf War to

non-deployers (n=3,695) found that deployers had significantly lower PCS and MCS scores (-2.1 and -1.9, respectively).(43) But this study did not separate out those who were injured while deployed, which is a risk factor for poor QOL. Our study examined personnel deployed to the more recent conflicts (OEF/OIF/OND) and found that those deployed and were not injured (DNI) had the highest QOL. These results corroborate previous research on the Healthy Warrior Effect that suggest deployed personnel have optimal health that protects them from later adverse mental and physical outcomes.(44-46) Our results suggest that any advantage deployers had prior to deployment may be attenuated if injured while deployed (either in battle or not).

Contrary to what was hypothesized, most of the variance in follow-up MCS and PCS was explained by baseline MCS and PCS scores. In addition, while having a minimal impact on the total variance for the fully adjusted models, some factors, including deployment and injury status, sleep duration, and smoking status, were found to have a strong and statistically significant association with QOL. Therefore, these factors could be explored in future research and used as potential intervention targets for service members or veterans.

This analysis had notable limitations. Participants were only included if they completed two surveys and survey completers may reflect a healthier subset of the original cohort. This may limit the generalizability of the findings to the cohort or the military overall. Also, since the study used non-injured as the reference groups, we could not account for injury factors such as injury severity, type, or cause. Despite these limitations, strengths of the analysis include higher statistical power because of Millennium Cohort Study's large sample size. In addition, inclusion into the Millennium Cohort Study was not conditional on injury status and therefore is more representative of the general military. In addition, the longitudinal design allowed for QOL to be assessed at two time points several years apart and measuring baseline covariates prior to the outcomes of interest allows for temporal associations to be examined.

While all groups' QOL worsened over time, certain groups had larger changes. Both constructs of QOL (mental and physical) worsened over time for injured service members, the difference in physical QOL reached a clinically meaningful difference for injured deployers (regardless of whether the injury occurred in battle or not) as compared to those who deployed and were not injured. This indicates that while prior QOL should be considered for research and practice, certain groups, such as those injured while deployed have poorer physical QOL longer-term outcomes than others, and thus should be considered as potential targets for physical, or vocational rehabilitation interventions.

Acknowledgments

In addition to the authors, the Millennium Cohort Study team includes Jennifer N. Belding, PhD; Satbir Boparai, MBA; Ania Bukowinski, MPH; Felicia Carey, PhD; Toni Rose Geronimo-Hara, MPH; Clinton Hall, MPH, PhD; David Ignacio; Isabel G. Jacobson, MPH; Cynthia A. LeardMann, MPH; Vanessa Perez, MPH; Aprilyn Piega; Anna Rivera, MPH; Rosa Salvatier; Neika Sharifian, MS, PhD; Beverly Sheppard; Steven Speigel; Daniel Trone, PhD; Javier Villalobos, MS; Jennifer Walstrom; and Katie Zhu, MPH. The authors also appreciate contributions from the Deployment Health Research Department, Millennium Cohort Family Study, Birth, and Infant Health Research Team, and Leidos, Inc. We greatly appreciate the contributions of the Millennium Cohort Study participants.

References

1. Rostker B. Providing for the Casualties of War: The American Experience Since World War II. Santa Monica, CA: RAND CORP 2020.
2. Howard JT, Kotwal RS, Stern CA, Janak JC, Mazuchowski EL, Butler FK, et al. Use of Combat Casualty Care Data to Assess the US Military Trauma System During the Afghanistan and Iraq Conflicts, 2001-2017. JAMA Surg. 2019;154(7):600-8.
3. Marklund N, (pp. 109-113). . Blast-Induced Brain Injury. Sundstrom T, Grände P-O, Luoto T, Rosenlund C, Undén J, Wester KG, editors. New York: Springer; 2020.
4. Tsai J, Whealin JM, Scott JC, Harpaz-Rotem I, Pietrzak RH. Examining the relation between combat-related concussion, a novel 5-factor model of posttraumatic stress symptoms, and health-related quality of life in Iraq and Afghanistan veterans. J Clin Psychiatry. 2012;73(8):1110-8.
5. Bilmes L. The Long-Term Costs of United States Care for Veterans of the Afghanistan and Iraq Wars. Providence, RI: Watson Institute for International and Public Affairs, Brown University; August 2021.
6. Moergeli H, Wittmann L, Schnyder U. Quality of life after traumatic injury: a latent trajectory modeling approach. Psychother Psychosom. 2012;81(5):305-11.
7. NEJM Catalyst. What Is Patient-Centered Care? ; January 1, 2017.
8. Wilson IB, Cleary PD. Linking clinical variables with health-related quality of life. A conceptual model of patient outcomes. Jama. 1995;273(1):59-65.
9. Carr AJ, Higginson IJ. Are quality of life measures patient centred? BMJ. 2001;322(7298):1357-60.
10. Epstein RA, Heinemann AW, McFarland LV. Quality of life for veterans and servicemembers with major traumatic limb loss from Vietnam and OIF/OEF conflicts. J Rehabil Res Dev. 2010;47(4):373-85.
11. Asano M, Rushton P, Miller WC, Deathe BA. Predictors of quality of life among individuals who have a lower limb amputation. Prosthet Orthot Int. 2008;32(2):231-43.
12. Singh JA, Borowsky SJ, Nugent S, Murdoch M, Zhao Y, Nelson DB, et al. Health-related quality of life, functional impairment, and healthcare utilization by veterans: veterans' quality of life study. J Am Geriatr Soc. 2005;53(1):108-13.
13. Vásquez PM, Durazo-Arvizu RA, Marquez DX, Argos M, Lamar M, Odoms-Young A, et al. Moderate-vigorous physical activity and health-related quality of life among Hispanic/Latino adults in the Hispanic Community Health Study/Study of Latinos (HCHS/SOL). J Patient Rep Outcomes. 2019;3(1):45.
14. Kyle SD, Morgan K, Espie CA. Insomnia and health-related quality of life. Sleep Med Rev. 2010;14(1):69-82.
15. Duffy JF, Zitting KM, Czeisler CA. The Case for Addressing Operator Fatigue. Rev Hum Factors Ergon. 2015;10(1):29-78.
16. Proctor S, Smith K, Heeren T, Vasterling J. Prospective Assessment of Health-Related Functioning Among Deployed U.S. Army Active Duty and National Guard Soldiers. Military Behavioral Health. 2014;2(1):42-51.
17. Staniute M, Brozaitiene J, Bunevicius R. Effects of social support and stressful life events on health-related quality of life in coronary artery disease patients. J Cardiovasc Nurs. 2013;28(1):83-9.

18. Woodruff SI, Galarneau MR, McCabe CT, Sack DI, Clouser MC. Health-related quality of life among US military personnel injured in combat: findings from the Wounded Warrior Recovery Project. Qual Life Res. 2018;27(5):1393-402.
19. Allison-Aipa TS, Ritter C, Sikes P, Ball S. The impact of deployment on the psychological health status, level of alcohol consumption, and use of psychological health resources of postdeployed U.S. Army Reserve soldiers. Mil Med. 2010;175(9):630-7.
20. Hoge CW, Auchterlonie JL, Milliken CS. Mental health problems, use of mental health services, and attrition from military service after returning from deployment to Iraq or Afghanistan. Jama. 2006;295(9):1023-32.
21. Smith TC, Millennium Cohort Study T. The US Department of Defense Millennium Cohort Study: career span and beyond longitudinal follow-up. J Occup Environ Med. 2009;51(10):1193-201.
22. Ryan MA, Smith TC, Smith B, Amoroso P, Boyko EJ, Gray GC, et al. Millennium Cohort: enrollment begins a 21-year contribution to understanding the impact of military service. J Clin Epidemiol. 2007;60(2):181-91.
23. Kauvar DS, Gurney J. Exploring Nonbattle Injury in the Deployed Military Environment Using the Department of Defense Trauma Registry. Mil Med. 2020;185(7-8):e1073-e6.
24. Huizinga E, Idenburg FJ, van Dongen T, Hoencamp R. Repatriation for diseases or non-battle injuries (DNBI): long-term impact on quality of life. BMJ Mil Health. 2020;166(E):e13-e6.
25. Kazis LE, Miller DR, Skinner KM, Lee A, Ren XS, Clark JA, et al. Patient-reported measures of health: The Veterans Health Study. J Ambul Care Manage. 2004;27(1):70-83.
26. Defense Health Agency. Joint Trauma System, The Department of Defense Center of Excellence for Trauma 2020 [Available from: https://jts.amedd.army.mil/index.cfm/data/registries). .
27. Hirshkowitz M, Whiton K, Albert SM, Alessi C, Bruni O, DonCarlos L, et al. National Sleep Foundation's sleep time duration recommendations: methodology and results summary. Sleep Health. 2015;1(1):40-3.
28. Smith B, Ryan MA, Wingard DL, Patterson TL, Slymen DJ, Macera CA. Cigarette smoking and military deployment: a prospective evaluation. Am J Prev Med. 2008;35(6):539-46.
29. Kroenke K, Wu J, Yu Z, Bair MJ, Kean J, Stump T, et al. Patient Health Questionnaire Anxiety and Depression Scale: Initial Validation in Three Clinical Trials. Psychosom Med. 2016;78(6):716-27.
30. American Psychiatric Association. Diagnostic and Statistical Manual of Mental Disorders: DSM-IV. Washington, DC Author; 1994.
31. Holmes TH, Rahe RH. The Social Readjustment Rating Scale. J Psychosom Res. 1967;11(2):213-8.
32. SAS Institute. The SAS system for Windows. Release 9.4. Cary, NC: SAS Institute; 2013.
33. McAndrew LM, D'Andrea E, Lu SE, Abbi B, Yan GW, Engel C, et al. What pre-deployment and early post-deployment factors predict health function after combat deployment?: a prospective longitudinal study of Operation Enduring Freedom (OEF)/Operation Iraqi Freedom (OIF) soldiers. Health Qual Life Outcomes. 2013;11:73.
34. Hoencamp R, Idenburg FJ, van Dongen TT, de Kruijff LG, Huizinga EP, Plat MC, et al. Long-term impact of battle injuries; five-year follow-up of injured Dutch servicemen in Afghanistan 2006-2010. PLoS One. 2015;10(2):e0115119.

35. van Dongen T, de Graaf J, Plat MJ, Huizinga EP, Janse J, van der Krans AC, et al. Evaluating the Military Medical Evacuation Chain: Need for Expeditious Evacuation Out of Theater? Mil Med. 2017;182(9):e1864-e70.
36. Kelly JF, Ritenour AE, McLaughlin DF, Bagg KA, Apodaca AN, Mallak CT, et al. Injury severity and causes of death from Operation Iraqi Freedom and Operation Enduring Freedom: 2003-2004 versus 2006. J Trauma. 2008;64(2 Suppl):S21-6; discussion S6-7.
37. Belmont PJ, Jr., McCriskin BJ, Sieg RN, Burks R, Schoenfeld AJ. Combat wounds in Iraq and Afghanistan from 2005 to 2009. J Trauma Acute Care Surg. 2012;73(1):3-12.
38. Galarneau MR, Woodruff SI, Dye JL, Mohrle CR, Wade AL. Traumatic brain injury during Operation Iraqi Freedom: findings from the United States Navy-Marine Corps Combat Trauma Registry. J Neurosurg. 2008;108(5):950-7.
39. Tomberg T, Toomela A, Pulver A, Tikk A. Coping strategies, social support, life orientation and health-related quality of life following traumatic brain injury. Brain Inj. 2005;19(14):1181-90.
40. Hauret KG, Pacha L, Taylor BJ, Jones BH. Surveillance of Disease and Nonbattle Injuries During US Army Operations in Afghanistan and Iraq. US Army Med Dep J. 2016(2-16):15-23.
41. Hauret KG, Taylor BJ, Clemmons NS, Block SR, Jones BH. Frequency and causes of nonbattle injuries air evacuated from operations iraqi freedom and enduring freedom, u.s. Army, 2001-2006. Am J Prev Med. 2010;38(1 Suppl):S94-107.
42. Sanders JW, Putnam SD, Frankart C, Frenck RW, Monteville MR, Riddle MS, et al. Impact of illness and non-combat injury during Operations Iraqi Freedom and Enduring Freedom (Afghanistan). Am J Trop Med Hyg. 2005;73(4):713-9.
43. Voelker MD, Saag KG, Schwartz DA, Chrischilles E, Clarke WR, Woolson RF, et al. Health-related quality of life in Gulf War era military personnel. American journal of epidemiology. 2002;155(10):899-907.
44. McLaughlin R, Nielsen L, Waller M. An evaluation of the effect of military service on mortality: quantifying the healthy soldier effect. Ann Epidemiol. 2008;18(12):928-36.
45. Larson GE, Highfill-McRoy RM, Booth-Kewley S. Psychiatric diagnoses in historic and contemporary military cohorts: combat deployment and the healthy warrior effect. American journal of epidemiology. 2008;167(11):1269-76.
46. Haley RW. Point: bias from the "healthy-warrior effect" and unequal follow-up in three government studies of health effects of the Gulf War. American journal of epidemiology. 1998;148(4):315-23.

Table 1: Demographic, behavioral, and military characteristics by injury status among Millennium Cohort participants, n=118,054

Variable	Not deployed (n=95,921) N (%)	Deployed and not injured (n=21,745) N (%)	Deployed and injured: Nonbattle (n=197) N (%)	Deployed and injured: Battle (n=191) N (%)
Time between baseline and follow-up surveys, in years (Mean, SD)	4.20 (2.22)	4.54 (1.62)	5.96 (3.29)	5.46 (3.03)
**MCS***				
Baseline (Mean, SD)	51.21 (10.24)	52.40 (8.58)	52.31 (8.85)	50.93 (10.10)
Change score (Mean, SD)	-1.43 (10.66)	-1.57 (10.20)	-3.35 (11.95)	-3.82 (13.20)
Follow-up (Mean, SD)	49.78 (11.27)	50.83 (10.08)	48.96 (12.36)	47.11 (13.00)
**PCS****				
Baseline (Mean, SD)	53.32 (7.84)	55.23 (5.97)	54.20 (6.36)	54.35 (6.68)
Change score (Mean, SD)	-1.13 (8.22)	-1.76 (7.68)	-8.80 (11.78)	-10.13 (11.78)
Follow-up (Mean, SD)	52.19 (8.49)	53.47 (7.39)	45.40 (11.48)	44.23 (11.05)
**Covariates**				
Age, in years (mean, SD)	32.56 (9.23)	29.27 (7.06)	29.19 (7.23)	28.16 (6.07)
Sex				
Male	64,736 (67.49)	16,704 (76.82)	162 (82.23)	178 (93.19)
Female	31,185 (32.51)	5,041 (23.18)	35 (17.77)	13 (6.81)
Race and Ethnicity				
Non-Hispanic Black	10,687 (11.14)	2,121 (9.75)	27 (13.71)	17 (8.90)
Non-Hispanic White	72,541 (75.63)	16,653 (76.58)	146 (74.11)	154 (80.63)
Other (including Hispanic, Asian/Pacific Islander, American/Alaskan Indian and other)	12,693 (13.23)	2,971 (13.66)	24 (12.18)	20 (10.47)
Marital Status				
Single	26,859 (28.00)	7,514 (34.56)	60 (30.46)	55 (28.80)
Married	57,222 (59.66)	12,240 (56.29)	112 (56.85)	125 (65.45)
Separated	11,840 (12.34)	1,991 (9.16)	25 (12.69)	11 (5.76)
Education				
Less than college	53,066 (55.32)	12,517 (57.56)	133 (67.51)	134 (70.16)
College degree	42,855 (44.68)	9,228 (42.44)	64 (32.49)	57 (29.84)
Component				
Reserve/ National Guard	39,583 (41.27)	6,656 (30.61)	54 (27.41)	56 (29.32)
Active duty	56,338 (58.73)	15,089 (69.39)	143 (72.59)	135 (70.68)
Service Branch				
Army	43,932 (45.80)	9,404 (43.25)	147 (74.62)	165 (86.39)
Navy or Coast Guard	18,966 (19.77)	2,777 (12.77)	13 (6.60)	3 (1.57)
Marine	7,934 (8.27)	1,326 (6.10)	15 (7.61)	14 (7.33)
Air Force	25,089 (26.16)	8,238 (37.88)	22 (11.17)	9 (4.71)
Pay Grade				
Enlisted	75,036 (78.23)	16,258 (74.77)	165 (83.76)	160 (83.77)
Officer	20,885 (21.77)	5,487 (25.23)	32 (16.24)	31 (16.23)
Number deployments (Mean, SD)	0.39 (0.85)	0.71 (1.32)	0.45 (0.70)	0.40 (0.64)
Average hours of sleep				
Less than 6 hours	17,734 (18.49)	3,861 (17.76)	49 (24.87)	45 (23.56)
6 hours	29,610 (30.87)	6,957 (31.99)	61 (30.96)	74 (38.74)
7-9 hours	45,535 (47.47)	10,440 (48.01)	79 (40.10)	65 (34.03)
More than 9 hours	3,042 (3.17)	487 (2.24)	8 (4.06)	7 (3.66)
Smoking status				
Never	56,002 (58.38)	13,470 (61.95)	107 (54.31)	94 (49.21)
Former	23,695 (24.70)	4,611 (21.20)	46 (23.35)	46 (24.08)
Current	16,224 (16.91)	3,664 (16.85)	44 (22.34)	51 (26.70)
Depression symptoms (yes)	4,209 (4.39)	465 (2.14)	6 (3.05)	7 (3.66)
PTSD symptoms (yes)	6,477 (6.75)	774 (3.65)	6 (3.05)	13 (6.81)
Life Stressors				
None	54,266 (56.57)	14,829 (68.19)	118 (59.90)	120 (62.83)
One	35,843 (26.94)	4,786 (22.01)	54 (27.41)	51 (26.70)
2 or more	15,812 (16.48)	2,130 (9.80)	25 (12.69)	20 (10.47)

MCS - Mental Composite Summary; PCS - Physical Composite Summary; MCS and PCS are derived from the Short-Form Health Survey for Veterans measure
*Mean MCS at baseline varied significantly by deployment and injury status (p-value < .0001). Pair wise comparisons using Tukey multiple comparison procedure revealed one significant (p-value < 0.05) group difference in the mean MCS at baseline between ND and DNI. Mean change in MCS between baseline and follow-up varied significantly by injury/deployment status (p-value < .0001). Pair wise comparisons using Tukey multiple comparison procedure revealed two significant (p-value < 0.05) group differences in the change in MCS between 1) DBI and ND, and 2) DBI and DNI. Mean MCS at follow-up varied significantly by injury/deployment status. Pair wise comparisons using Tukey multiple comparison procedure revealed three significant differences (p-value < 0.05) in mean MCS at follow-up between 1) ND and DNI, 2) DBI and DNI, and 3) DBI and ND.
** Mean PCS scores at baseline varied significantly by deployment and injury status (p-value < .0001). Pair wise comparisons using Tukey multiple comparison procedure revealed one significant (p-value < 0.05) group difference in the mean PCS between those DNI and ND. Mean change in PCS between baseline and follow-up varied significantly by injury/deployment status (p-value < .0001). Pair wise comparisons using Tukey multiple comparison procedure revealed significant (p-value < 0.05) group differences in the change in PCS between all groups except those DNBI and DBI. Mean PCS at follow-up varied significantly by injury/deployment status. Pair wise comparisons using Tukey multiple comparison procedure revealed significant difference (p-value < 0.05) in mean PCS at follow-up between all comparisons except between DNBI and DBI.
Smoking status – defined as never and former/current based off of responses to questions regarding smoking at least 100 cigarettes in their lifetime and successfully quitting smoking cigarettes.
Depression symptoms – assessed using the PHQ depression scale (PHQ-8) based on DSM-IV-TR criteria. Participants screened positive for depression based on the following criteria: responded "more than half the days" or "nearly every day" to at least 5 items with at least one item endorsed being depressed mood or anhedonia.
PTSD symptoms – assessed using the PTSD Checklist – Civilian Version. The measure consists of 17 items which evaluate PTSD symptoms during the previous month. A positive PTSD screen was determined based using the criteria as defined in the Diagnostic and Statistical Manual of Mental Disorders, Fourth Edition, Text Revision.
Life stressors – based on the number of endorsed stressful life experiences in the past 3 years including endorsement of divorce, financial problems, physical assault, sexual assault, and illness or death of a loved.

Table 2: Associations between main exposure and covariates with Mental Composite Scores (MCS) among Millennium Cohort participants, n=118,054

Variable	Model 1 Estimate (SE)	Model 2 Estimate (SE)	Model 3 Estimate (SE)
**Baseline MCS**	0.554 (0.003) **	0.553 (0.003)**	0.468 (0.004)**
**Baseline PCS**			0.174 (0.004)**
**Injury Category (ref: Deployed and not injured)**			
Deployed and battle injured		-2.904 (0.698)**	-1.863 (0.677)*
Deployed and nonbattle injured		-1.817 (0.687)*	-0.829 (0.667)
Not deployed		-0.388 (0.072)**	-0.321 (0.073)**
**Time between surveys, in years**			-0.254 (0.013)**
**Age (5-year change)**			0.552 (0.019)**
**Female sex (ref: Male)**			-0.467 (0.064)**
**Race and Ethnicity (ref: Non-Hispanic White)**			
Non-Hispanic Black			0.981 (0.091)**
Other (including Hispanic, Asian/Pacific Islander, American/Alaskan Indian and other)			-0.172 (0.081)*
**Marital Status* (ref: Single)**			
Married			0.121 (0.068)
Separated			0.233 (0.105)*
**College Degree Education* (ref: Some college or less)**			0.013 (0.070)
**Reserve/National Guard Component* (ref: Active Duty)**			-0.442 (0.061)**
**Service Branch* (ref: Army)**			
Navy/Coast Guard			0.507 (0.077)**
Marine			-0.348 (0.108)*
Air Force			1.611 (0.068)**
**Officer Pay Grade* (ref: Enlisted)**			0.447 (0.083)**
**Number Deployments (cumulative)**			-0.528 (0.029)**
**Average hours of sleep (ref: 7-9 hours)**			
< 6 hours			-0.866 (0.082)**
6 hours			-0.382 (0.064)**
9 < hours			-0.619 (0.164)*
**Smoking status (ref: never)**			
Former			-0.439 (0.068)**
Current			-0.618 (0.079)**
**Depression symptoms (ref: no)**			-0.472 (0.170)*
**PTSD symptoms (ref: no)**			-2.152 (0.145)**
**Life Stressors (ref: none)**			
One			-0.464 (0.069)**
2 or more			-1.287 (0.090)**
**R^2**	0.248	0.248	0.294
**Root MSE**	9.605	9.603	9.308

* <0.05 ** <0.0001

Table 3: Associations between main exposure and covariates with Physical Composite Scores (PCS) among Millennium Cohort participants, n=118,054

Variable	Model 1 Estimate (SE)	Model 2 Estimate (SE)	Model 3 Estimate (SE)
**Baseline PCS**	0.526 (0.003)**	0.525 (0.003)**	0.476 (0.003)**
**Baseline MCS**			0.077 (0.003)**
**Injury Category (ref: Deployed and not injured)**			
Deployed and battle injured		-8.784 (0.532)**	-7.937 (0.516)**
Deployed and nonbattle injured		-7.529 (0.524)**	-6.580 (0.508)**
Not deployed		-0.278 (0.055)**	-0.162 (0.055)*
**Time between surveys, in years**			-0.386 (0.010)**
**Age (5-year change)**			-0.448 (0.015)**
**Female sex (ref: Male)**			-0.109 (0.049)*
**Race and Ethnicity (ref: Non-Hispanic White)**			
Non-Hispanic Black			-0.314 (0.070)**
Other (including Hispanic, Asian/Pacific Islander, American/Alaskan Indian and other)			-0.112 (0.062)
**Marital Status* (ref: Single)**			
Married			-0.366 (0.052)**
Separated			-0.454 (0.080)**
**College Degree Education* (ref: Some college or less)**			0.441 (0.054)**
**Reserve/National Guard Component* (ref: Active Duty)**			0.758 (0.047)**
**Service Branch* (ref: Army)**			
Navy/Coast Guard			1.079 (0.059)**
Marine			0.498 (0.082)**
Air Force			0.622 (0.052)**
**Officer Pay Grade* (ref: Enlisted)**			0.941 (0.063)**
**Number Deployments (cumulative)**			-0.391 (0.022)**
**Average hours of sleep (ref: 7-9 hours)**			
< 6 hours			-0.960 (0.062)**
6 hours			-0.307 (0.049)**
9 < hours			-0.101 (0.125)
**Smoking status (ref: never)**			
Former			-0.231 (0.052)**
Current			-0.601 (0.060)**
**Depression symptoms (ref: no)**			0.041 (0.130)
**PTSD symptoms (ref: no)**			-0.308 (0.110)*
**Life Stressors (ref: none)**			
One			-0.390 (0.053)**
2 or more			-1.025 (0.069)**
**R^2**	0.227	0.230	0.276
**Root MSE**	7.330	7.315	7.095

* <0.05 ** <0.0001

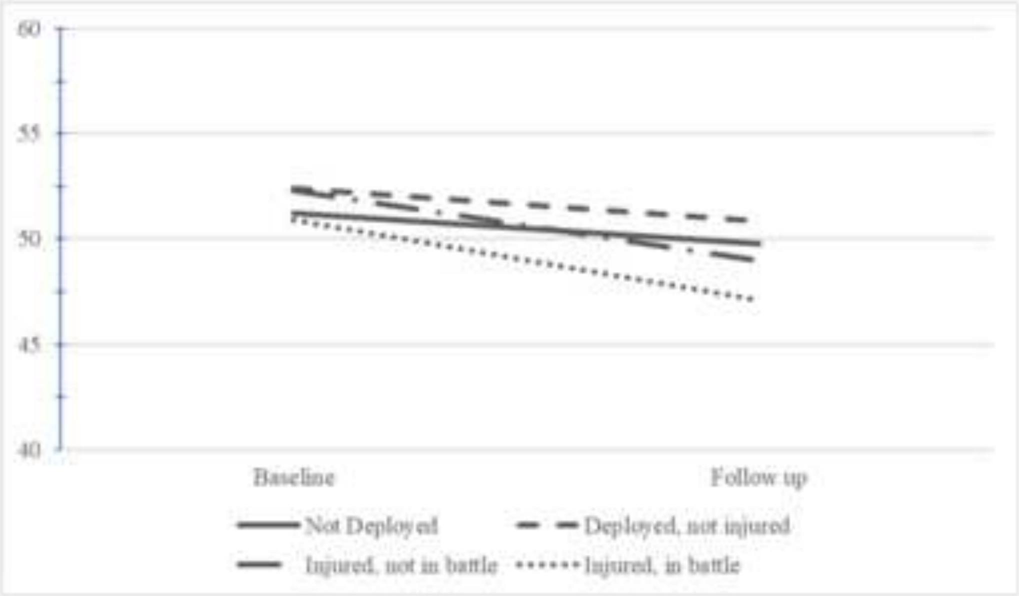

Figure 1: Average MCS by deployment and injury status at baseline and follow-up. Mean MCS at baseline varied by deployment and injury status (mean of 51.2 for ND, 52.4 for DNI, 52.3 for DNBI, and 50.9 for DBI; p-value < .0001; Table 1 and Figure 2) and pairwise comparisons using Tukey multiple comparison procedures revealed a significant (p-value < 0.05) group difference between ND and DNI's mean scores. Mean change in MCS between baseline and follow-up also varied significantly by deployment and injury status (mean change for ND -1.4, DNI -1.6, DNBI -3.4, and DBI -3.8; p-value < .0001; Table 1 and Figure 2). Pairwise comparisons revealed two significant (p-value < 0.05) group differences in the change in MCS between those DBI and the two non-injured groups. Finally, mean MCS at follow-up varied significantly by deployment and injury status (Table 1 and Figure 2) and pairwise comparisons revealed three significant differences (p-value < 0.05) in mean MCS at follow-up between 1) ND and DNI, 2) DNI and DBI, and 3) ND and DBI.

Click here to access/download;Figure;Figure2_2FEB2022.tif

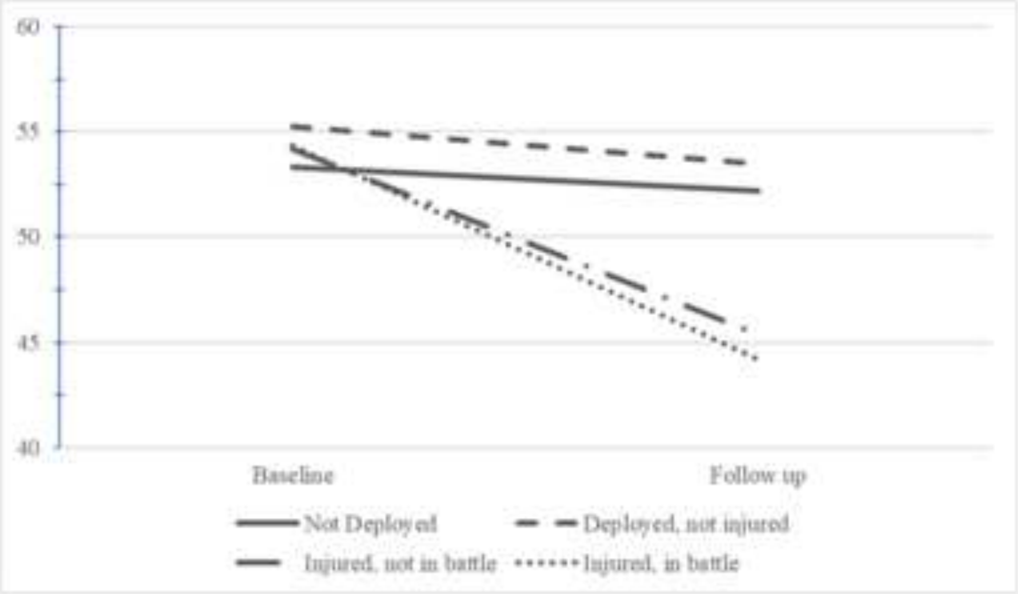

Figure 2: Average PCS by deployment and injury status at baseline and follow-up. Mean PCS at baseline varied by deployment and injury status (mean of 53.3 for ND, 55.2 for DNI, 54.2 for DNBI, and 54.4 for DBI; p-value < .0001; Table 1 and Figure 2) and pairwise comparisons revealed a significant (p-value < 0.05) group difference between ND and DNI's mean baseline PCS. Mean change in PCS between baseline and follow-up varied significantly by deployment and injury status (mean change for ND -1.1, DNI -1.8, DNBI -8.8, and DBI -10.1; p-value < .0001; Table 1 and Figure 2) with significant (p-value < 0.05) group differences found between all groups except the two injured groups. Additionally, mean PCS at follow-up varied significantly by deployment and injury status (Table 1 and Figure 2) where pairwise comparisons again revealed significant difference (p-value < 0.05) between all groups except between the two injured groups.

Conflict of Interest

Click here to access/download
Supporting Information
CoI_7MAR2022.docx
